# Mitral re-valve-in-valve as a new perspective for high-risk patients with prosthetic valve dysfunction: case reports

**DOI:** 10.1093/ehjcr/ytad579

**Published:** 2023-11-23

**Authors:** Matheus Ramos Dal Piaz, Lucas Tachotti Pires, Jonathan Cayo Urdiales Herrera, André Luis Bezerra Labat, Felipe Reale Cividanes, Guilherme Sobreira Spina, José Honório Palma, Flávio Tarasoutchi

**Affiliations:** Faculdade de Medicina, Instituto do Coração, Hospital das Clínicas HCFMUSP, Universidade de São Paulo, 05403-000 São Paulo, SP, Brazil; Faculdade de Medicina, Instituto do Coração, Hospital das Clínicas HCFMUSP, Universidade de São Paulo, 05403-000 São Paulo, SP, Brazil; Faculdade de Medicina, Instituto do Coração, Hospital das Clínicas HCFMUSP, Universidade de São Paulo, 05403-000 São Paulo, SP, Brazil; Faculdade de Medicina, Instituto do Coração, Hospital das Clínicas HCFMUSP, Universidade de São Paulo, 05403-000 São Paulo, SP, Brazil; Faculdade de Medicina, Instituto do Coração, Hospital das Clínicas HCFMUSP, Universidade de São Paulo, 05403-000 São Paulo, SP, Brazil; Faculdade de Medicina, Instituto do Coração, Hospital das Clínicas HCFMUSP, Universidade de São Paulo, 05403-000 São Paulo, SP, Brazil; Faculdade de Medicina, Instituto do Coração, Hospital das Clínicas HCFMUSP, Universidade de São Paulo, 05403-000 São Paulo, SP, Brazil; Faculdade de Medicina, Instituto do Coração, Hospital das Clínicas HCFMUSP, Universidade de São Paulo, 05403-000 São Paulo, SP, Brazil

**Keywords:** Mitral valve prosthesis, Transcatheter valve implantation, Prosthesis failure, Case reports

## Abstract

**Background:**

Mitral valve diseases are a common medical condition, and surgery is the most used therapeutic approach. The need for less invasive interventions led to the development of transcatheter valve implantation in high-risk patients. However, the treatment to the dysfunctions of these prosthetic valves is still uncertain, and the yield and safety of repeated transcatheter valve implantations remain unclear.

**Cases summary:**

A 69-year-old Caucasian woman with three previous mitral valve procedures performed due to rheumatic valve disease (currently with a biological prosthetic mitral valve) and a 76-year-old Latin woman with previous liver transplantation (due to metabolic-associated fatty liver disease) and biological mitral prosthesis due to mitral valve prolapse with severe regurgitation underwent mitral valve-in-valve (ViV) transcatheter implantation at the time of dysfunction of their surgical prostheses. Later, these patients developed prosthetic valve dysfunction and clinical worsening, requiring another invasive procedure. Due to maintained high-risk status and unfavourable clinical conditions for surgery, re-valve-in-valve (re-ViV) was performed.

**Discussion:**

Valve-in-valve transcatheter mitral valve implantation was approved in 2017, and, since then, it has been used in several countries, mainly in high-risk patients. Nevertheless, these prosthetic valves may complicate with stenosis or regurgitation, demanding reinterventions. Although there are favourable data for mitral ViV, re-ViV still lacks robust data to support its performance, with only case reports in the literature so far. It is possible that in high-risk patients, there is a greater benefit from re-ViV when compared with the surgical strategy. However, this hypothesis must be studied in future controlled trials.

**Learning points**

- Prosthetic mitral valves may complicate with both stenosis and regurgitation, demanding reinterventions.
- In high-risk patients, the treatment of prosthetic valve complications with re-valve-in-valve is feasible and secure.

## Introduction

Mitral valve diseases are a common medical condition^1^ that can lead to structural cardiac complications and have high morbidity. Surgical treatment of the mitral valve has been the most used therapeutic approach. However, technological advances and the need for less invasive interventions enabled the development of transcatheter valve implantation, which has also been applied in the treatment of mitral valve diseases.^2^

Biological valve prostheses have an average durability of ∼15 years. The standard treatment in case of dysfunction is reoperation, despite its association with higher morbidity and mortality.^3^ In patients with high surgical risk, the mitral valve-in-valve (ViV) has been adopted as a less invasive alternative, with lower incidence of complications.^4^ The safety and efficacy of this procedure have been demonstrated in previous trials with positive outcomes as acceptable early mortality and improvement in patient symptoms.^5,6^ However, information about the treatment to the dysfunctions of these new prosthetic valves remains scarce in literature.

This article reports two patients who underwent mitral re-valve-in-valve (re-ViV): the transcatheter mitral valve implantation (TMVI) to treat the mechanical dysfunction of a previous transcatheter mitral prosthetic valve. The procedures were performed using the prosthesis Inovare (Braile®, Sao Jose do Rio Preto, Brazil) with transapical access.

## Summary figure

**Table:** 

Patient 1	
24 years ago	Surgical mitral valve replacement with a mechanical prosthesis.
20 years ago	The mechanical prosthesis was replaced by a biological prosthesis.
6 years ago	Mitral ViV due to stenosis of the biological prosthesis.
Day 0	Patient was admitted with dyspnoea and clinical signs of heart failure due to prosthetic valve stenosis.
Day 21	Re-ViV using a transapical access.
Day 31	Patient was discharged 10 days after the procedure with improved symptoms and without complications.
4 years later	Patient remained asymptomatic with no limitations [New York Heart Association (NYHA) functional Class I].
Patient 2	
10 years ago	Surgical mitral valve replacement with a biological prosthesis due to mitral regurgitation.
10 months ago	Mitral ViV due to prosthetic dysfunction (stenosis).
Day 0	Patient was admitted with dyspnoea on minimal exertion associated with clinical signs of heart failure and haemolytic anaemia.
Day 8	Sepsis by *Klebsiella pneumoniae* complex.
Day 34	Re-ViV using a transapical access due to displacement of the prosthetic mitral valve with periprosthetic regurgitation.
Day 37	Sepsis by *Serratia marcescens*.
Day 47	Infection in the right femoral region.
Day 48	Transoesophageal echocardiography showed thrombus in one of the leaflets of the new biological prosthetic valve.
Day 115	Patient died of septic shock.

## Case summary

### Patient 1

A 65-year-old Caucasian woman was admitted with exertional limiting dyspnoea (NYHA functional Class IV) and clinical signs of decompensated heart failure (central cyanosis, tachypnoea—35 breaths per minute). Her physical examination showed opening snap of the mitral valve and a mid-diastolic rumble. The B-type natriuretic peptide (BNP) was 179 pg/mL. The patient had a record of atrial fibrillation and prior ischaemic stroke of cardioembolic aetiology, with a long-term right-sided hemiparesis. Due to rheumatic mitral valve disease, she previously had three mitral valve procedures. The first one was in 1995, when the mitral valve was replaced by a mechanical prosthesis. The second one was in 1999, this time the first mechanical prosthesis was replaced by a biological one. And the last procedure was in 2013, when a stenosis of the biological prosthesis was diagnosed and the mitral ViV was performed with the implantation of an Inovare prosthetic valve (Braile®, Number 26). At that time, the patient already had high surgical risk and unfavourable clinical conditions for a new sternotomy. In 2019, at hospital admission, she had functional limitations due to dyspnoea and the sequelae of her previous ischaemic stroke with Katz index of 3 and a Lawton-Brody index of 1.^7,8^

**Figure 1:**
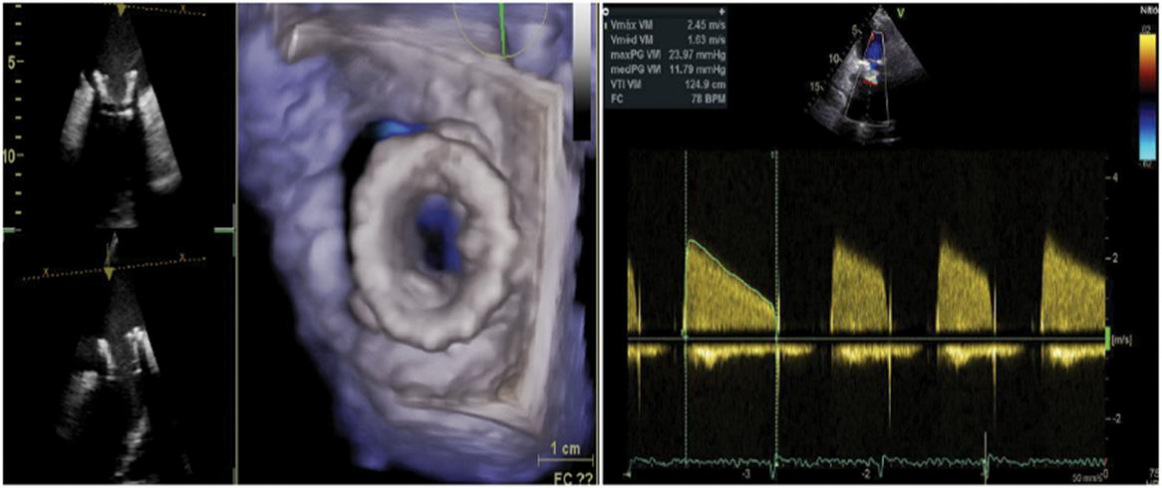
Pre-procedural echocardiogram shows prosthetic valve stenosis with a maximum and mean mitral transvalvular diastolic gradient of 24 and 12 mmHg, respectively [60.9 × 148 mm (×DPI)].

In transoesophageal echocardiography, there was no mobility of the anterolateral and posterior leaflets of the prosthetic mitral valve (only the anteromedial leaflet presented good mobility). The valve area was 0.6 cm^2^ by planimetry and 0.7 cm^2^ by pressure half-time (PHT), with maximum and mean mitral diastolic gradients of 23 and 12 mmHg, respectively (*Figure 1*). The left ventricle ejection fraction was 64%, the right ventricle had mild systolic dysfunction, the pulmonary artery systolic pressure was 70 mmHg, and the tricuspid valve had moderate regurgitation.

**Figure 2:**
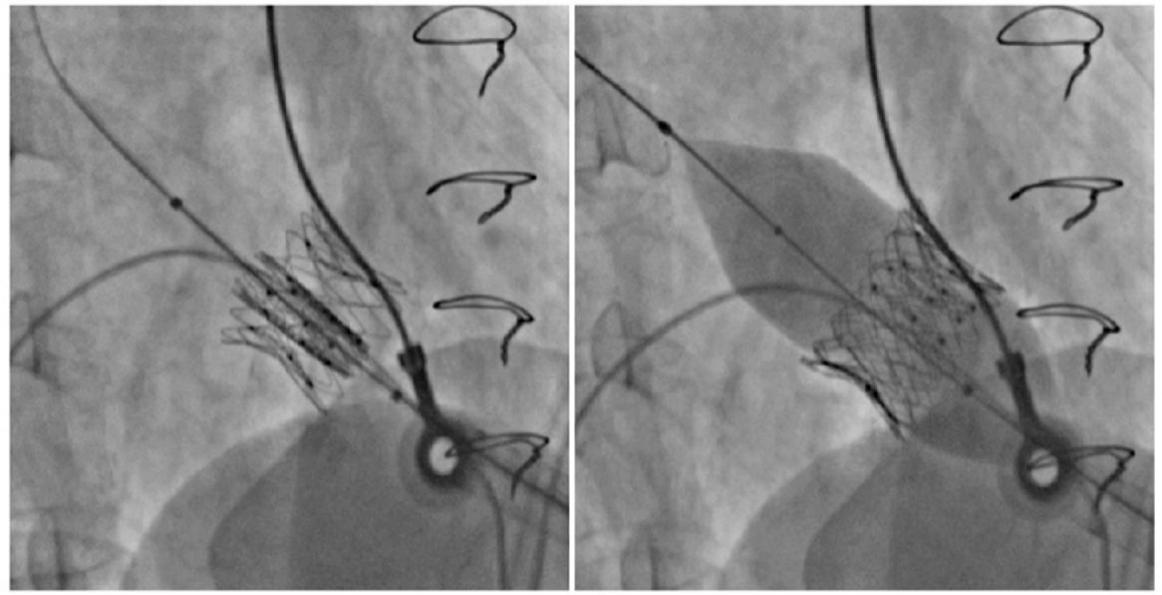
Fluoroscopy: transcatheter heart valve positioning and implantation [75.4 × 147 mm (×DPI)].

**Figure 3:**
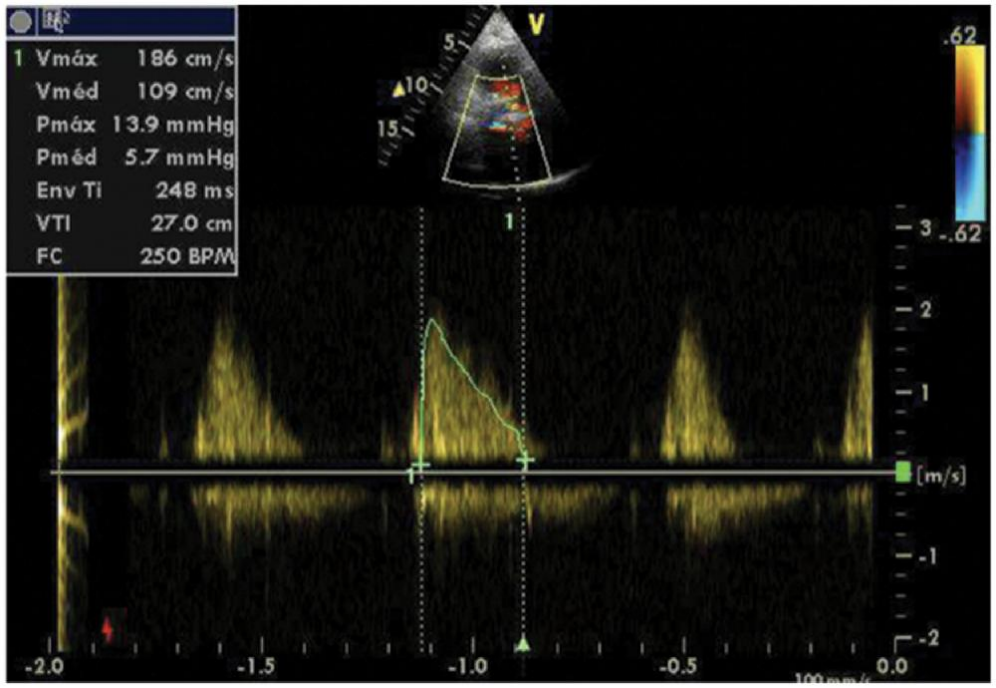
Post-procedural echocardiogram reveals a significant reduction in transprosthetic pressure gradient (from 12 to 5 mmHg) and a well-placed valve [91.5 × 133 mm (×DPI)].

After discussing with the heart team the increased surgical risk (EuroSCORE II 5.49%) associated with clinical frailty of the patient, it was decided to perform re-ViV with implantation of an Inovare prosthetic valve (Braile®, Number 24) using transapical access (*Figure 2*). Once the patient had already undergone prior transapical ViV and adhesions of the pericardium could be present, a greater caution was required for the dissections and there were no organic lesions. The postoperative echocardiography revealed minimal central regurgitation of the prosthetic valve, with maximum and mean mitral diastolic gradients of 13 and 5 mmHg, respectively, and an estimated valve area of 1.8 cm^2^ using 3D planimetry (*Figure 3*). The patient was discharged from the hospital 10 days after the procedure with improved symptoms and without complications. Since her first follow-up visit 1 month after the discharge, the patient remained asymptomatic without limitations (NYHA functional Class I) and still is nowadays, 4 years after the procedure.

### Patient 2

**Figure 4:**
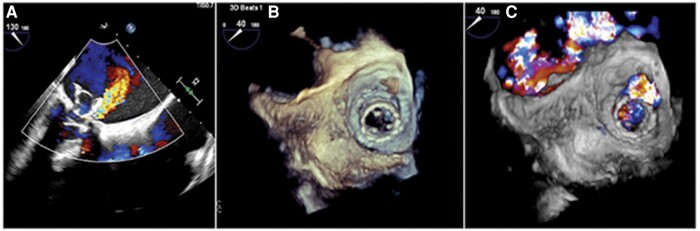
Sequence of pre-procedural echocardiogram images showing displacement of the prosthetic mitral valve towards the left atrium with a significant periprosthetic regurgitation. (*A*) Transthoracic echocardiogram Doppler colour image. (*B* and *C*) 3D echocardiogram [51.0 × 159 mm (×DPI)].

A 76-year-old Latin woman with previous liver transplantation in 2012, systemic arterial hypertension, Parkinson’s disease, and chronic obstructive pulmonary disease underwent mitral valve replacement with a biological prosthesis in 2012 (Inovare biological prosthesis, Braile®, Number 29) due to mitral regurgitation that was caused by mitral valve prolapse. As the patient had high risk for a new cardiac surgery, in 2021, after having diagnosed stenosis of the biological prosthesis, she underwent ViV (Inovare biological prosthesis, Braile®, Number 26). After ViV, echocardiography showed the prosthesis had normal opening of its leaflets, an effective orifice area of 1.62 cm^2^, a mean gradient of 7 mmHg, and mild-to-moderate paravalvular leak. The procedure resulted in progressive improvement of her symptoms during the following 8 months, from NYHA functional Class III to I. However, 10 months after ViV, she was admitted with dyspnoea on minimal exertion (NYHA functional Class IV), haemolytic anaemia, and clinical signs of heart failure (tachypnoea—24 breaths per minute, bilateral lung wheezing, prolonged expiration, and oedema of the lower limbs). Her physical examination showed frequent extrasystoles and a regurgitant systolic murmur. Blood tests revealed BNP 135 pg/mL, haemoglobin 7.2 g/dL, indirect bilirubin 1.48 mg/dL, haptoglobin < 0.08 g/L, reticulocyte count 12.1%, and lactate dehydrogenase 2155 UI/L. Transthoracic echocardiography showed a prosthetic mitral valve with a mean diastolic gradient of 6 mmHg and severe periprosthetic regurgitation (between the surgical prosthesis and the first ViV prosthesis) with a regurgitant orifice of 9 × 5 mm and an effective regurgitant orifice area of 0.4 cm^2^. Transoesophageal echocardiography demonstrated displacement of the prosthetic mitral valve towards the left atrium, with projection of its stem towards the left ventricle outflow tract (LVOT) and a LVOT gradient of 25 mmHg. Such displacement was probably associated with partial recoil of the Inovare biological prosthesis and was responsible for the periprosthetic regurgitation (*Figure 4*, Supplementary material online, *Videos S1* and *S2*), leading to the signs and symptoms of heart failure and haemolytic anaemia. The left ventricle ejection fraction was 60%, the right ventricle had normal systolic function, the pulmonary artery systolic pressure was 46 mmHg, and the tricuspid valve had moderate regurgitation. At the time of hospital admission, her symptoms were causing functional impairment and the patient needed help for basic activities of daily life, presenting Katz index of 3 and a Lawton–Brody index of 1.^7,8^

During hospitalization, she developed acute renal failure that required renal replacement therapy and also developed urinary and bloodstream infection by *K. pneumoniae* complex. After clinical compensation and antibiotic therapy, the heart team decided for mitral re-ViV considering the patient remained with a high surgical risk (EuroSCORE II 16.68%).

**Figure 5:**
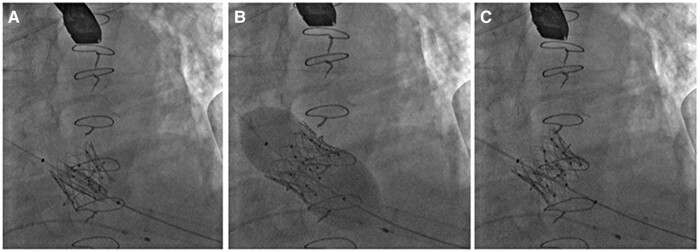
Fluoroscopy (*A*) shows overlapping of the valve stent struts prior to dilatation. (*B*) During the procedure, a balloon was used to upsize the stent struts. (*C*) Final angiographic result [57.5 × 163 mm (×DPI)].

**Figure 6:**
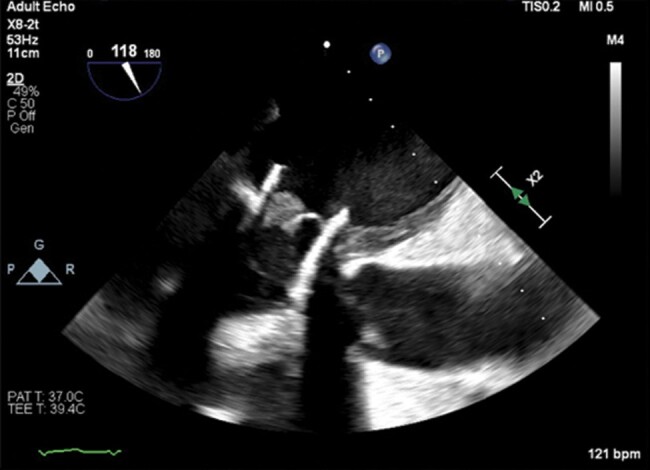
Transoesophageal echocardiogram reveals a dilated left atrium with an isoechoic mass straddling the leaflets of the new biological prosthetic valve [94 × 129 mm (×DPI)].

She underwent re-ViV with Inovare biological prosthesis (Braile®, Number 28) using transapical access (*Figure 5*). The possible risk of complete embolization of the previous prosthesis due to the manipulation of the catheters represented a great technical challenge during the procedure. Despite this concern, the surgical team succeeded in the implantation of the new prosthetic valve without immediate complications. The intraoperative echocardiography demonstrated no periprosthetic regurgitation and a decrease in the LVOT gradient from 25 to 18 mmHg immediately after the delivery of the new prosthesis (Supplementary material online, *Videos S3* and *S4*). However, in the first days after the procedure, the patient had an infection in the right femoral region and later in the bloodstream by *S. marcescens*. She also developed an inguinal lymphatic fistula. These complications developed where the right femoral artery and vein were prepared by dissection as stand-by accesses to perform a possible on-pump cardiac surgery. Two weeks after the procedure, transoesophageal echocardiography showed thickening of one of the leaflets of the new prosthetic valve, suggestive of a thrombus (*Figure 6*) with maximum and mean mitral diastolic gradients of 23 and 12 mmHg, respectively. In this exam, there was no more LVOT gradient and no periprosthetic regurgitation. Anticoagulation with intravenous heparin was started with full reversal of the alterations previously found. However, the patient was debilitated by recurrent infections that were not associated with the preprocedural ones and died of septic shock 81 days after re-ViV.

## Discussion

Nowadays, there is a great interest in the new percutaneous techniques used for the treatment of valvulopathies. The revolution in the therapeutic approach of aortic stenosis in the elderly due to technological advances and the excellent results obtained with the transcatheter aortic valve implantation (TAVI) paved the way for a less invasive treatment of the mitral and tricuspid valves. In this context, the ViV technique, which consists of the transcatheter implantation of a biological prosthesis, has been used as a therapeutic option for high-risk patients who have dysfunction of the surgical bioprosthesis in the aortic or mitral valve position.^2^

There are three possible types of TMVI that can be used for high-risk patients: ViV for patients with severe dysfunction of a mitral valve prosthesis, valve-in-ring for a new mitral valve dysfunction after valve repair with a prosthetic ring, and valve-in-mitral annulus calcification (MAC) for the cases of mitral valve dysfunction due to severe MAC. The Food and Drug Administration approved mitral ViV for high-risk patients in 2017, while the possible other indications are still being evaluated by the agency.^9^

After the promising results with prosthetic aortic valve implantation, the first mitral ViV transcatheter implantation was published in 2009 by the Vancouver group, followed by a series of other publications confirming its technical viability.^10^

Patients with mitral valve diseases are often younger, with only 10% of people over 75 years old being affected by mitral valve regurgitation in developed countries. Consequently, TMVI treatment occurs in younger subjects than those treated with TAVI.^3^

A meta-analysis published in 2023 in the *American Journal of Cardiology* described that mitral ViV was associated with better outcomes than a new surgery, including lower rates of complications and shorter length of hospital stay. However, there was no significant difference in mortality rate.^11^ The 30-day mortality rate for ViV went from 8.8% back in 2014 to 3.9% in 2019.^12^ Since 2014, the transapical approach has gradually decreased from 76 to 3.8% of the procedures.^9,13^ This is because the transseptal approach allows a less invasive procedure with less morbidity during hospitalization. For the treatment of the patients described, the Braile Inovare system was used, once it is the only prosthesis routinely available in our institution for the mitral ViV procedure. As the delivery system of this prosthesis only permits the use of the transapical approach, this was the access route chosen for our patients. To minimize procedural risk during re-ViV, some strategies could be applied, as performing the surgery in a hybrid operation room and having all the material available to perform on-pump surgery if needed, including intraoperative cell savage.

Considering that re-ViV is a new procedure, not yet widely performed, any decision in its favour should involve shared decision-making with the patient. In the presented cases, the treatment options were offered for the patients with disclosure of all risks and involved limitations. For example, possible complications of the transcatheter mitral prosthesis implantation are the obstruction of the LVOT and patient–prosthesis mismatch. Therefore, it was extensively explained for the patients the importance of adequate pre-procedure tomographic study for better planning of the procedure and to avoid these complications. The choice of re-ViV was eventually defined as the result of a common agreement between the heart team and the preferences of patients.

Although previous studies have indicated favourable results for mitral ViV, the re-ViV still lacks robust data to support its performance. So far, it only has case reports in literature. As an example, Khan *et al.*^12^ recently reported a similar case involving a 39-year-old woman with a record of two previous surgeries and a mitral ViV that underwent a successfully re-ViV with a SAPIEN 3 bioprosthesis. In their report, even though the Society of Thoracic Surgeons risk score was not elevated, it was considered that there was an increased surgical risk for the patient because of two prior sternotomy procedures and a complex medical history. Similarly, in our cases, the selection of both patients for the re-ViV procedure was guided by the association of increased surgical risk, clinical frailty, and repeated previous invasive interventions. Alternative approaches were discussed for each patient, such as transcatheter plug placement for Patient 2. However, after an extensive analysis, this approach was considered too risky for this patient due to the possibility of migration of the plug itself once the paravalvular leak was located between the two prostheses (surgical and first ViV).

## Conclusion

It is possible that in high-risk patients such as the ones presented in this study, there is a greater clinical net benefit from re-ViV when compared with surgical strategy. However, this hypothesis must be further studied in interventional randomized controlled trials. Likewise, as illustrated in this paper, this procedure may be useful in stenotic or regurgitant prosthesis dysfunction, and future trials are needed to identify the predictors of success to establish patient candidacy for re-ViV.

## Supplementary Material

ytad579_Supplementary_Data

## Data Availability

The data underlying this article will be shared upon reasonable request to the corresponding author.

## Lead author biography

Dr Matheus Dal Piaz is a cardiology resident at the Heart Institute of the University of Sao Paulo Medical School, São Paulo. He graduated in 2019, at the medical school of the University of Espírito Santo, and has finished internal medicine at the State University of Campinas, in 2022. He has specific interest in valvular heart diseases and arrhythmias.

## Supplementary material

**Consent:** Informed consent was obtained from Patient 1 and provided by the daughter of Patient 2 in accordance with COPE guidelines.

**Funding:** None declared.
